# Nitrogen availability and genotype affect major nutritional quality parameters of tef grain grown under irrigation

**DOI:** 10.1038/s41598-020-71299-x

**Published:** 2020-08-31

**Authors:** Zipora Tietel, Ella Simhon, Kelem Gashu, Devanesan Arul Ananth, Betty Schwartz, Yehoshua Saranga, Uri Yermiyahu

**Affiliations:** 1 Food Science, Agricultural Research Organization, Gilat Research Center, 8531100 M.P. Negev, Israel; 2 Institute of Biochemistry, Food Science and Nutrition, Faculty of Agricultural, Food and Environmental Quality Sciences, The Hebrew University of Jerusalem, P.O. Box 12, 76100 Rehovot, Israel; 3 Robert H. Smith Institute of Plant Sciences and Genetics in Agriculture, The Hebrew University of Jerusalem, P.O. Box 12, 76100 Rehovot, Israel; 4 Soil Chemistry, Plant Nutrition and Microbiology, Agricultural Research Organization, Gilat Research Center, 8531100 M.P. Negev, Israel

**Keywords:** Plant sciences, Biochemistry

## Abstract

Worldwide demand for tef (*Eragrostis tef*) as a functional food for human consumption is increasing, thanks to its nutritional benefits and gluten-free properties. As a result, tef in now grown outside its native environment in Ethiopia and thus information is required regarding plant nutrition demands in these areas, as well as resulting grain health-related composition. In the current work, two tef genotypes were grown in Israel under irrigation in two platforms, plots in the field and pots in a greenhouse, with four and five nitrogen treatments, respectively. Nutritional and health-related quality traits were analyzed, including mineral content, fatty acid composition, hydrophilic and lipophilic antioxidative capacity, total phenolic content and basic polyphenolic profile. Our results show that tef genotypes differ in their nutritional composition, e.g. higher phenolic contents in the brown compared to the white genotype. Additionally, nitrogen availability positively affected grain fatty acid composition and iron levels in both experiments, while negatively affecting total phenolics in the field trials. To conclude, nitrogen fertilization is crucial for crop growth and productivity, however it also implicates nutritional value of the grains as food. These effects should be considered when fertilizing tef with nitrogen, to optimize both crop productivity and nutritional effects.

## Introduction

Tef (*Eragrostis tef*) is an annual tropical Poaceae cereal, grown in a wide range of environments, and used throughout the world as grain for human consumption^1^. Tef is indigenous to Ethiopia^2^, where it is a staple food for majority of Ethiopians and for millions of people in the semi-arid regions of the world^1^.

From a nutritional point of view, tef is naturally gluten free^3,4^, with a low glycemic index^5^. It is rich in important minerals such as iron (Fe), calcium (Ca), potassium (K) and zinc (Zn)^6–10^ as well as in copper (Cu), phosphorous (P) and magnesium (Mg)^11^. Tef also contains remarkable levels of vitamin C, niacin, vitamin A, riboflavin and thiamin^12,13^. It has an average of 10.4% protein, 2.3% fat and 3.3% fiber^12,13^ and is rich in oleic and linoleic acid^14^, unsaturated essential fatty acids. Tef protein has an excellent amino acid composition, including all 8 essential amino acids and relatively high in lysine^15^. These data suggest that tef is as nutritious as or better than the major western staple cereals such as wheat, rice, oats and barley. It is preferred by celiac disease patients as it is consumed as a whole grain, and thus provides much higher nutritional value compared to other components of gluten-free diets^16^. It was also suggested to be incorporated into bread made of other cereal, to increase their health benefits^17,18^ and sensory quality^19^.

Furthermore, tef is rich in phenolic acids and flavonoids, which consumption was correlated with lower incidence of chronic diseases (diabetes, cardiovascular, cancer) possibly thanks to their bioactivity as antioxidants and anti-inflammatory^20^. Tef genotypes vary in color from purple through red-brown to ivory white^21^, with dark tef considered to have the highest antioxidative potential accompanied by highest phenolic and flavonoids contents, although this might be genotype dependent^22^. Hence, tef is gaining enormous popularity in the international health food market, due to its attractive nutritional profile and its gluten free property^4,11^.

Owing to the high diversity between tef cultivars^23^, genotype might have a major impact on processing, eating and nutritional quality of food products^24^. However, knowledge about effect of genetic background on genotypes' nutritional composition and health benefits as well as processing and eating properties is limited, as most of the available studies only relied on one or two genotypes^24,25^.

The chemical composition of cereals varies greatly both between and within species and depends on the environmental conditions, soil, genotype and fertilizer^9^. Research indicates that application of nitrogen (N), phosphorus (P), potassium (K) and sulfur (S) fertilizers generally increases nutritional quality^26^. Specifically, increasing availability of N may increase the contents of some secondary metabolites such as carotenes and phenols in various crops^27–29^.

Nevertheless, not much work has been done on plant nutrition and fertilization in tef. Several works have shown the importance of N and P fertilization, increasing both dry matter and grain yields^30–32^. A genetic variation was reported between modern and landraces genotypes in N-use efficiency (defined by the authors as a ratio of grain yield to N-fertilizer supply), which was related to higher total grain N and total plant N in genotypes with higher uptake efficiency at low N-supply^33^. N use efficiency was also affected by soil type, N fertilization form and timing. Sulfur and N fertilization were also shown to increase S and N contents of the grains^30,31,34^. In addition, cultivars may vary in P uptake^35^, which may affect their yield^33,36^. Tef showed negative nutrient balance for N and K compared to enset (*Ensete ventricosum*)-based systems^37^, and Zn was reported as a yield limiting factor for tef^38^. None of these works did however evaluate the effect on grain health-related properties.

At the same time, from a nutritional point of view, N fertilization in high rates may reduce the accumulation of defense-related secondary metabolites and vitamin C levels^39,40^, in addition to dietary fibers and Mg and Ca levels, while increasing the levels of carotenes and vitamin B1^26,39,40^. Additionally, high N fertilization generally led to a decrease in anthocyanin and flavonoid content, resulting also in lower antioxidative capacity^41^. Thus, optimization of the fertilization practice is important in maximizing the nutritional values of tef.

The aim of the current work was to study the effect of N fertilization on tef nutritional traits, in order to further develop N fertilization practices to improve the nutritional value of tef. The study combined tef response to N in commercial-like (field) condition with greenhouse (pot) condition. In addition, we wanted to study, for the first time, the phytochemical and nutritional composition of tef grown in Israel.

## Results

### Mineral content

Table 1 presents the mineral content range for the white and brown tef grains grown in field and pot experiments. Our results show that 100 gr dry weight (DW) of white tef grains contained 169–226 mg Ca, 184 to 195 mg Mg, 8.47 to 8.95 mg Fe, 4.43 to 7.42 mg Zn, 5.49 to 9.32 mg Mn and 0.2 1to 0.94 mg Cu (Table 1). Brown tef grains contained 173 to 232 mg Ca, 188 to 231 mg Mg, 9.39 to 10.57 mg Fe, 4.03 to 8.07 mg Zn, 5.67 to 15.33 mg Mn and 0.22 to 0.84 mg Cu for 100 gr DW (Table 1). Compared to the brown tef, white tef had higher levels of Mg, Zn and Cu in the field experiment, while the brown genotype had higher levels of Mn in the pot experiment (Table 1).

**Table 1:** Average values (± standard error (S.E.)) for nutritional quality parameters in white and brown tef genotypes grown in the field and pot.

	Field			Pot		
	White variety (406W)	Brown variety (405B)	*P* value	White variety (406W)	Brown variety (405B)	*P* value
Ca (mg/100 gr DW)	226.1 ± 4.8	232.7 ± 4.7		169.2 ± 8.2	173.3 ± 8.6	
Mg (mg/100 gr DW)	195.5 ± 1.8	188.2 ± 1.8	*	188.2 ± 8.5	209.4 ± 9.0	
Fe (mg/100 gr DW)	8.5 ± 0.5	9.4 ± 0.5		8.9 ± 3.03	10.6 ± 3.2	
Zn (mg/100 gr DW)	4.43 ± 0.04	4.03 ± 0.04	*	7.42 ± 0.44	8.07 ± 0.46	
Mn (mg/100 gr DW)	5.49 ± 0.09	5.67 ± 0.1		9.32 ± 0.46	15.3 ± 0.5	*
Cu (mg/100 gr DW)	0.94 ± 0.01	0.84 ± 0.01	*	0.21 ± 0.03	0.22 ± 0.04	
C14:0 (%)	0.03 ± 0	0.03 ± 0		0.03 ± 0	0.03 ± 0	
C15:0 (%)	0.02 ± 0	0.02 ± 0		0.02 ± 0	0.02 ± 0	
16:0 (%)	17.0 ± 0.14	17.3 ± 0.14		15.7 ± 0.14	15.3 ± 0.14	*
C16:1 (Z7) (%)	0.04 ± 0	0.04 ± 0		0.03 ± 0	0.03 ± 0	
C16:1 (Z9) (%)	0.19 ± 0.01	0.19 ± 0.01		0.13 ± 0.01	0.11 ± 0.01	*
C17:0 (%)	0.1 ± 0	0.09 ± 0		0.19 ± 0.03	0.18 ± 0.03	
C18:0 (%)	5.5 ± 0.05	5.2 ± 0.05	*	4.0 ± 0.06	3.6 ± 0.06	*
18:11 (Z9) (%)	27.5 ± 0.1	26.6 ± 0.1	*	26.2 ± 0.2	26.8 ± 0.2	*
C18:1 (Z11) (%)	0.70 ± 0.01	0.72 ± 0.01	*	0.62 ± 0.01	0.60 ± 0.01	
C18:2 (%)	41.7 ± 0.1	42.4 ± 0.11	*	46.6 ± 0.2	46.3 ± 0.2	
C18:3 (%)	5.2 ± 0.03	5.6 ± 0.03	*	5.3 ± 0.05	6.0.0 ± 0.05	*
C20:0 (%)	1.13 ± 0.03	0.94 ± 0.03	*	0.68 ± 0.01	0.6 ± 0.01	*
C20:1 (Z11) (%)	0.2 ± 0.01	0.21 ± 0.01		0.21 ± 0.01	0.23 ± 0.01	*
C20:1 (Z13) (%)	0.36 ± 0.01	0.33 ± 0.01	*	0.14 ± 0.01	0.18 ± 0.01	
C21:0 (%)	0.05 ± 0	0.05 ± 0		0.04 ± 0	0.05 ± 0	
C22:0 (%)	0.29 ± 0.01	0.22 ± 0.01	*	0.30 ± 0.01	0.20 ± 0.01	
SFA (%)	24.1 ± 0.1	23.9 ± 0.1		20.7 ± 0.1	19.7 ± 0.1	*
MUFA (%)	28.9 ± 0.1	28.2 ± 0.1	*	27.2 ± 0.2	28.0 ± 0.2	*
PUFA (%)	47.0 ± 0.1	48.0 ± 0.1	*	51.8 ± 0.2	52.3 ± 0.2	*
HAC (µmoleTE/mg DW)	3.26 ± 0.12	3.22 ± 0.12		1.28 ± 0.07	1.17 ± 0.07	
LAC (µmoleTE/mg DW)	1.73 ± 0.08	1.52 ± 0.08		2.0 ± 0.11	2.66 ± 0.11	*
Phenolics -free (mg RE/g DW)	2.24 ± 0.04	2.14 ± 0.04		2.02 ± 0.08	2.02 ± 0.08	
Phenolics-bound (mg RE/g DW)	0.73 ± 0.07	0.93 ± 0.07		0.25 ± 0.05	0.37 ± 0.04	
TPC (mg GAE/gr DW)	1.2 ± 0.02	1.27 ± 0.02	*	0.89 ± 0.05	1.04 ± 0.05	*
Genistic acid (µg/g DW)	88.6 ± 18.8	126.1 ± 19.1		19.5 ± 2.4	19.7 ± 2.6	
Vanillin (µg/g DW)	37.9 ± 9.8	174.7 ± 9.9	*	16.5 ± 10.1	157.6 ± 10.4	*
p-coumaric acid (µg/g DW)	46.0 ± 5.2	19.5 ± 5.2	*	165.4 ± 24.0	3.92 ± 25.26	*
Ferulic acid (µg/g DW)	79.6 ± 6.9	126.1 ± 7.04	*	19.3 ± 3.3	75.9 ± 3.2	*
Rutin (µg/g DW)	146.3 ± 14.1	125.6 ± 14.2		262.9 ± 13.6	189.1 ± 14.3	*
Quercetin (µg/g DW)	1,403.7 ± 131.9	1,429.5 ± 134.1		1,607.8 ± 109.8	1,486.2 ± 115.4	

Asterisk (*) stands for *p* < 0.05, for the specific *p* values please see Table 2 (for field results) or Table 3 (for pot results).

**Figure 1:**
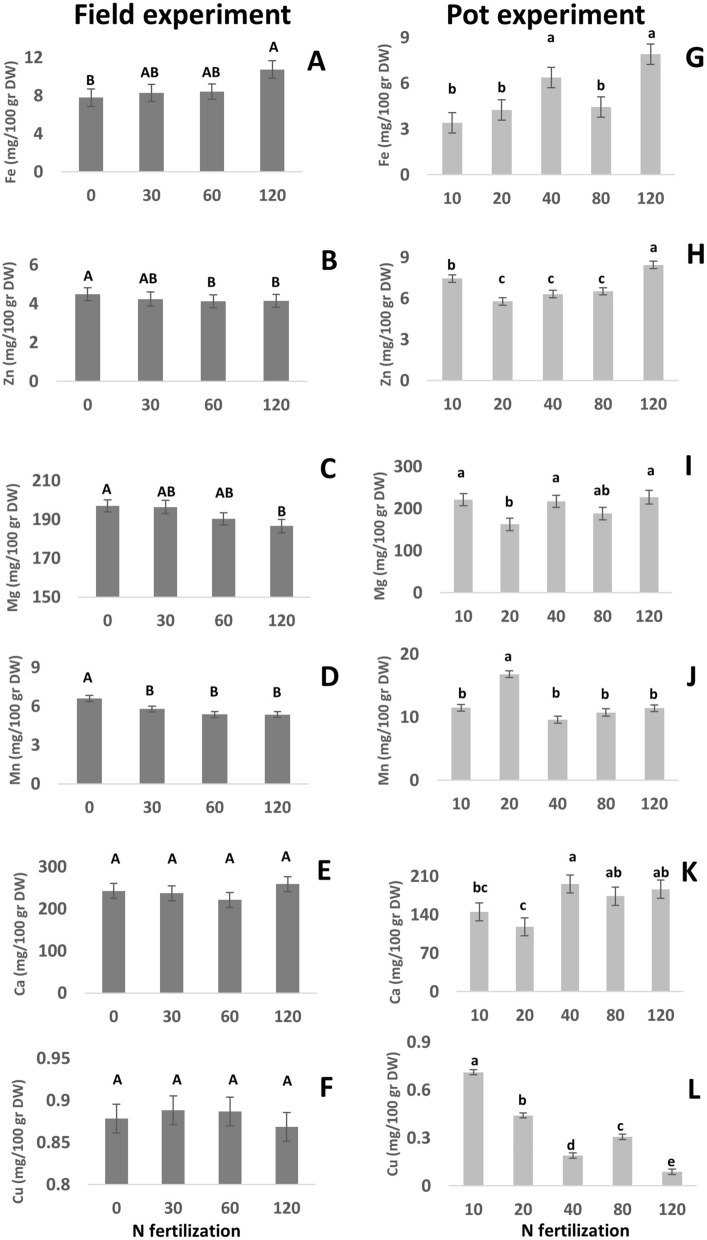
Effect of nitrogen (N) treatments on mineral content of tef grains, in field (dark bars, **A**–**F**) and pot (light bars, **G**–**L**) experiments. All figure results are presented as mean ± standard error (SE).

Tables 2 and 3 present the statistical analysis of the response of nutritional quality attributes in tef to N fertilization in the field and pot experiments, respectively. Field experiment results show that tef grain mineral levels were significantly affected by N fertilization. An increase was observed in Fe levels with increasing N, from 7.75 mg/100 gr DW in 0 ppm N to 10.7 mg/100 gr DW in 120 ppm N (Fig. 1A). A decrease was recorded in Zn levels, which were higher in 0 ppm (4.5 mg/100 gr DW) compared to in 60 and 120 ppm (4.15 and 4.12 mg/100 gr DW, respectively; Fig. 1B). as well as in Mg levels, decreasing from 197 mg/100 gr DW at 0 ppm N to 187 mg/100 gr DW at 120 ppm (Fig. 1C). Mn levels also decreased from 6.61 mg/100 g DW at 0 ppm to 5.78, 5.37 and 5.35 in 30, 60 and 120 ppm, respectively) (Fig. 1D). Ca and Cu levels did not change in the field experiment in response to N fertilization (Fig. 1E,F, respectively).

**Table 2:** Statistical analysis parameters for the response of nutritional quality attributes of tef grains to N fertilization in the field experiment.

	Model F value	*P* value interaction	*P* value N	*P* value variety	Intercept	Slope N	R^2^
Ca (mg/100 gr DW)	0.524	0.398	0.734	0.331	227.3	0.046	0.066
Mg (mg/100 gr DW)	0.017	0.802	0.021	0.006	197.5	− 0.09	0.348
Fe (mg/100 gr DW)	0.064	0.898	0.024	0.205	7.50	0.024	0.263
Zn (mg/100 gr DW)	0.0003	0.078	0.023	0.0001	4.38	− 0.003	0.537
Mn (mg/100 gr DW)	0.014	0.185	0.004	0.158	6.25	− 0.009	0.346
Cu (mg/100 gr DW)	0.0001	0.65	0.573	0.0001	0.89	− 0.0001	0.585
C14:0 (%)	0.874	0.586	0.663	0.498	0.03	− 0.00002	0.013
C15:0 (%)	0.087	0.568	0.029	0.688	0.02	− 0.00005	0.227
C16:0 (%)	0.01	0.873	0.018	0.132	17.6	− 0.0087	0.4
C16:1 (Z7) (%)	0.906	0.653	0.758	0.76	0.043	− 0.00001	0.009
C16:1 (Z9) (%)	0.824	0.298	0.622	0.997	0.185	0.0001	0.018
C17:0 (%)	0.653	0.851	0.439	0.265	0.099	− 0.0001	0.04
C18:0 (%)	0.326	0.668	0.604	0.014	5.41	− 0.0007	0.101
C18:1 (Z9) (%)	0.57	0.782	0.81	0.0001	27.16	− 0.0006	0.452
C18:1 (Z11) (%)	0.044	0.914	0.036	0.019	0.699	0.0003	0.28
C18:2 (%)	0.017	0.509	0.017	0.0003	41.6	0.0077	0.335
C18:3 (%)	0.007	0.645	0.144	0.0001	5.35	0.0017	0.378
C20:0 (%)	0.018	0.788	0.442	0.0001	1.09	− 0.0005	0.331
C20:1 (Z13) (%)	0.386	0.908	0.256	0.199	0.204	− 0.0002	0.1
C20:1 (Z11) (%)	0.569	0.798	0.406	0.027	0.357	− 0.0002	0.058
C21:0 (%)	0.126	0.417	0.054	0.147	0.051	− 0.0002	0.196
C22:0 (%)	0.088	0.584	0.369	0.0002	0.279	− 0.0003	0.226
SFA (%)	0.027	0.556	0.008	0.251	24.43	− 0.0084	0.304
MUFA (%)	0.009	0.783	0.824	0.0001	28.66	− 0.0007	0.362
PUFA (%)	0.004	0.48	0.006	0.0001	46.84	0.0095	0.438
HAC (µmoleTE/mg DW)	0.114	0.704	0.039	0.799	3.824	− 0.0098	0.187
LAC (µmoleTE/mg DW)	0.176	0.954	0.089	0.097	1.212	0.005	0.175
Phenolics-free (mg RE/g DW)	0.582	0.408	0.614	0.056	2.261	− 0.0005	0.055
Phenolics-bound (mg RE/g DW)	0.236	0.523	0.158	0.055	1.09	− 0.003	0.128
TPC (mg GAE/gr DW)	0.016	0.81	0.011	0.034	1.302	− 0.0015	0.369
Genistic acid (µg/g DW)	0.24	0.647	0.104	0.168	58.97	0.664	0.155
Vanillin (µg/g DW)	0.0002	0.375	0.084	0.0001	66.11	0.455	0.598
p-coumaric acid (µg/g DW)	0.002	0.138	0.003	0.0006	8.782	0.462	0.483
Ferulic acid (µg/g DW)	0.004	0.837	0.62	0.0001	112.2	− 0.097	0.44
Rutin (µg/g DW)	0.042	0.988	0.017	0.305	202.6	− 0.924	0.284
Quercetin (µg/g DW)	0.009	0.185	0.004	0.894	1989.5	− 8.13	0.469

All table results are presented as mean ± SE.

**Table 3:** Statistical analysis parameters for the response of nutritional quality attributes of tef grains to N fertilization in the pot experiment.

	Model F value	*P* value interaction	*P* value N	*P* value variety	Intercept	Slope N	R^2^
Ca (mg/100 gr DW)	0.05	0.11	0.02	0.73	143.50	0.49811	0.20
Mg (mg/100 gr DW)	0.98	0.29	0.87	0.10	197.46	0.03351	0.00
Fe (mg/100 gr DW)	0.01	0.95	0.00	0.72	3.85	0.02922	0.29
Zn (mg/100 gr DW)	0.01	0.15	0.01	0.34	6.17	0.01364	0.32
Mn (mg/100 gr DW)	< 0.0001	0.90	0.17	0.00	13.87	− 0.01991	0.53
Cu (mg/100 gr DW)	< 0.0001	0.91	< .0001	0.94	0.58	− 0.00422	0.62
C14:0 (%)	0.75	0.15	0.48	0.18	0.03	0.00002	0.03
C15:0 (%)	0.03	0.81	0.02	0.55	0.01	0.00005	0.31
16:0 (%)	0.06	0.19	0.03	0.02	15.05	0.00954	0.28
C16:1 (Z7) (%)	0.91	0.42	0.85	0.49	0.03	0.00000	0.01
C16:1 (Z9) (%)	0.86	0.33	0.82	0.01	0.11	0.00003	0.02
C17:0 (%)	0.20	0.74	0.08	0.70	0.29	− 0.00153	0.16
C18:0 (%)	0.02	0.73	0.54	0.00	3.89	− 0.00090	0.37
18:11 (Z9) (%)	0.90	0.84	0.71	0.02	26.17	− 0.00184	0.01
C18:1 (Z11) (%)	0.90	0.24	0.76	0.27	0.60	− 0.00007	0.01
C18:2 (%)	0.65	0.25	0.36	0.26	46.94	− 0.00336	0.05
C18:3 (%)	< 0.0001	0.95	0.79	0.0001	5.76	0.00037	0.71
C20:0 (%)	0.00	0.32	0.02	0.0001	0.60	0.00081	0.56
C20:1 (Z11) (%)	0.12	0.93	0.17	0.002	0.18	− 0.00027	0.24
C20:1 (Z13) (%)	0.27	0.86	0.12	0.20	0.24	− 0.00027	0.16
SFA (%)	0.00	0.11	0.02	0.73	19.87	0.00792	0.54
MUFA (%)	0.95	0.72	0.77	0.39	27.24	0.00139	0.01
PUFA (%)	0.10	0.68	0.49	0.43	52.70	− 0.00299	0.23
HAC (µmoleTE/mg DW)	0.40	0.32	0.26	0.28	1.34	− 0.00133	0.07
LAC (µmoleTE/mg DW)	0.44	0.91	0.21	0.0001	2.21	0.00601	0.06
Phenolics-free (mg RE/g DW)	0.09	0.11	0.18	1.00	2.28	− 0.00114	0.18
Phenolics-bound (mg RE/g DW)	0.04	0.94	0.19	0.07	0.16	0.00169	0.66
TPC (mg GAE/gr DW)	0.01	0.88	0.02	0.014	0.84	0.00223	0.29
Genistic acid (µg/g DW)	0.71	0.89	0.42	0.96	25.65	− 0.05076	0.03
Vanillin (µg/g DW)	< 0.0001	0.90	0.08	0.0001	109.87	− 0.37473	0.74
p-coumaric acid (µg/g DW)	0.02	0.96	0.61	0.0001	46.83	0.24071	0.27
Ferulic acid (µg/g DW)	< 0.0001	0.10	0.82	0.0001	47.95	− 0.01662	0.79
Rutin (µg/g DW)	< 0.0001	0.54	0.01	0.0002	220.16	0.50297	0.72
Quercetin (µg/g DW)	0.75	0.78	0.46	0.44	1,772.93	0.82448	0.03

All table results are presented as mean ± SE.

Pot experiment showed a significant increase in Fe from 3.4, 4.3 and 4.4 mg/100 gr DW in 10, 20 and 80 ppm N, to 7.1 and 7.9 in 40 and 120 ppm, respectively (Fig. 1G). Zn contents increased with N fertilization, from 7.5, 5.8, 6.3 and 6.5 mg/100 gr DW in 10, 20, 40 and 80 ppm to 8.4 at 120 ppm (Fig. 1H). Mg levels were only slightly affected, with lower level of 162.4 mg/100 gr DW in 20 ppm, compared to 221.3, 217.1, 188.1 and 227.0 mg/100 gr DW in 10, 40, 80 and 120 ppm, respectively (Fig. 1I), and same trend was observed for Mn, with only one treatment (20 ppm–16.8 mg/100 gr DW) higher than others (11.5, 9.6, 10.7 and 11.4 mg/100 gr DW in 10, 40, 80 and 120 pp, respectively; Fig. 1J). Ca contents increased with N fertilization, from 118 mg/100 gr DW at 20 ppm N to 196, 173.8 and 186.4 mg/100 gr DW at 40, 80 and 120 ppm, respectively (Fig. 1K), while a decrease was observed in Cu content from 0.09 to 0.71 mg/100 gr DW in 120 and 0 ppm, respectively (Fig. 1L).

### Fatty acid composition

In this work we present a very detailed profile of tef fatty acids, with results being in agreement with other available data^42^, and show for the first time the fatty acid (FA) composition of brown vs. white tef grains. Genotypes showed differences in their fatty acid composition, which although being small were significant. Fatty acid composition comprised of C18:2 as the main fatty acid in both the white (41.7–46.6%) and the brown (42.4–46.3%) genotypes, followed by C18:1 Z9 (26.2–27.5% and 26.6–26.8% in white and brown genotypes, respectively), and C16:0 (15.7–17.0%-15.3–17.3%) (Table 1). These were followed by C18:3 (5.2–5.3% and 5.6–6.0%) C18:0 (4.0–5.5% and 3.6–5.2%) and C20:0 (0.68–1.13% and 0.6–0.94%). The FA C18:1 Z11 (0.62–0.70 and 0.60–0.72%), C20:1 Z11 (0.21–0.36% and 0.23–0.33%), C17:0 (0.1–0.19% and 0.09–0.18%), C16:1 Z9 (0.13–0.19% and 0.11–0.19%), C20:1 Z13 (0.14–0.2% 0.18–0.21%), C22:0 (0.22 and 0.29%), were all minor fatty acids. C14:0, C15:0, C16:1 Z7 and C21:0 were all below 0.1%. Total saturated fatty acids (SFA) (20.7–24.1% and 19.7–23.9%), monounsaturated fatty acids (MUFA) (27.2–28.9% and 28.0–28.2%) and polyunsaturated fatty acids (PUFA) (47.0–51.8% and 48.0–52.3%) reflected the FA composition of tef.

The tested tef cultivars greatly varied in their fatty acid composition: the white genotype contained higher levels of C18:0, C18:1Z9, C20:0, C20:1 Z11, C22:0 and MUFA, and the brown was higher in C18:1(Z11), C18:2, C18:3 and PUFA in the field. In the pot experiment, the white genotype was higher in the levels of C16:0, C16:1 Z9, C18:0, C20:0 and SFA while the brown was higher in C18:1 Z9, C18:3, C20:1 Z11, MUFA and PUFA.

**Figure 2:**
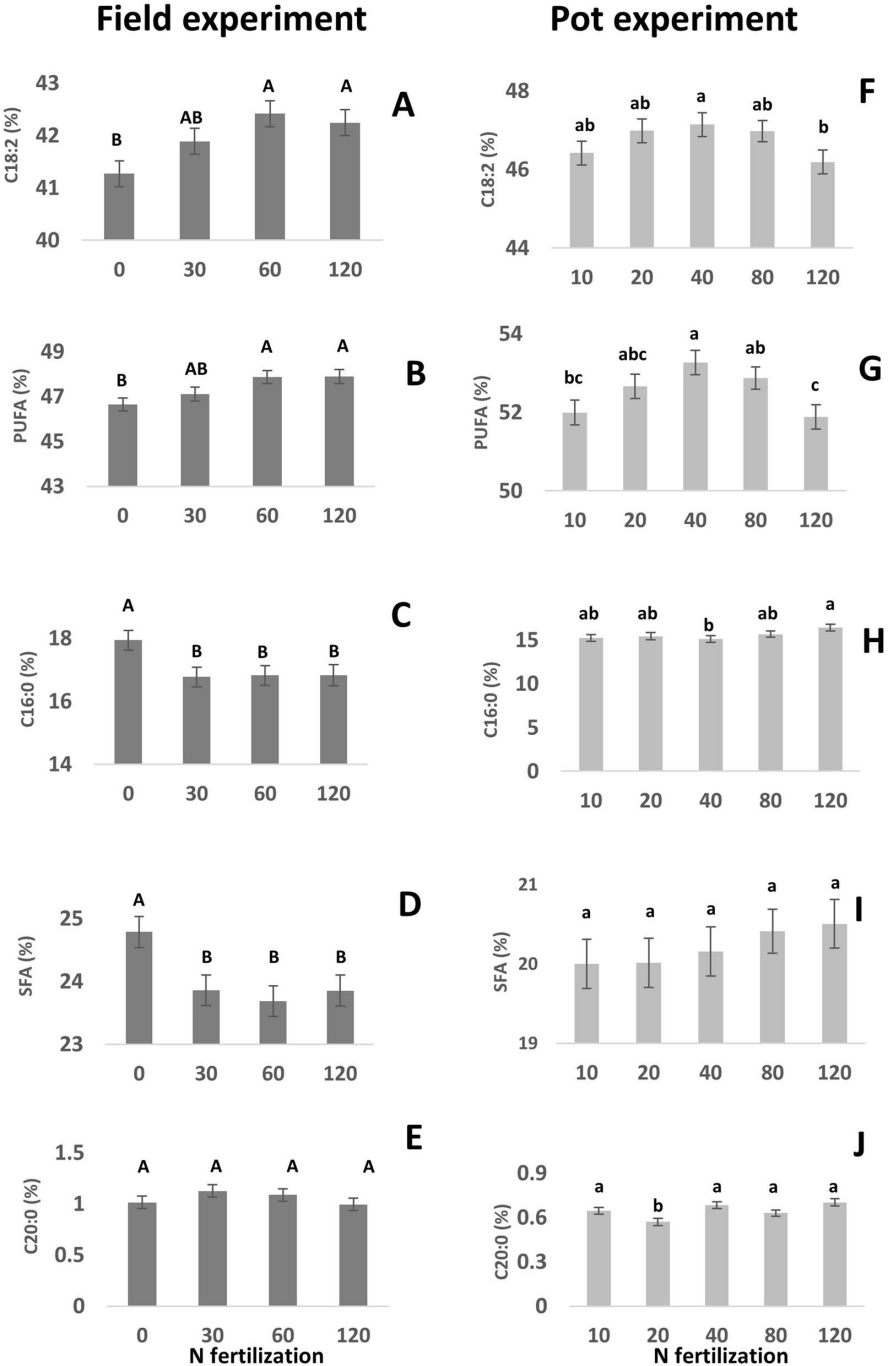
Effect of nitrogen (N) availability on fatty acid composition of tef grains, in field (dark bars; **A**–**E**) and pot (light bars; **F**–**J**) experiments. All figure results are presented as mean ± SE.

Fatty acid composition of tef was widely affected by N fertilization in both field and pot experiments. In the field, a significant increase was recorded in C18:2 with increasing N, from 41.3% in 0 ppm to 42.4% and 42.3% in 60 and 120 ppm, respectively (Fig. 2A), as well as in C18:1(Z11) levels, from 0.69% in 0 ppm to 0.73% in 120 ppm (data not shown). Total PUFA contents also increased, from 46.6% in 0 ppm to 47.9% in both 60 and 120 ppm N (Fig. 2B). At the same time, a decrease was observed in contents of C16:0 (17.9% in 0 ppm to 16.8% in 30, 60 and 120 ppm; Fig. 2C), and C15:0 (0.024% to 0.016% and 0.017% in 0 ppm vs. 30 and 60 ppm, respectively; data not shown), as well as in total SFA, from 24.8% in 0 ppm to 23.8 in 30, 60 and 120 ppm (Fig. 2D). C20:0 fatty acid was not affected (Fig. 2E).

In pots, levels of C18:2 were higher in 40 ppm treatment (47.1%) compared to 120 ppm (46.2%; Fig. 2F), while C18:1 (Z11) content was not affected by N fertilization (data not shown). PUFA levels were also higher in 40 ppm compared to 120 ppm and 10 ppm (53.3%, 51.9% and 52.0%, respectively; Fig. 2G). On the contrary, the levels of C16:0 were higher in 120 ppm compared to 40 ppm (16.4% and 15.1%, respectively; Fig. 2H), while levels of C15:0 increased with increasing N fertilization, from 0.01% at 0 ppm to 0.019% at 120 ppm (data not shown). SFA levels were not affected by N fertilization (Fig. 2I), and C20:0 levels were low only at 20 ppm (0.57%; Fig. 2J).

### Antioxidative capacity

In this work, we used the method described earlier by Vinokur et al. to analyze both hydrophilic antioxidative capacity (HAC) and lipophilic antioxidative capacity (LAC) of the same sample^79^. HAC of tef grains varied between 1.28–3.26 µmole TE/mg DW in the white genotype and 1.17–3.22 µmole TE/mg DW, with the brown genotype showing higher capacity than the white in the pot experiment. LAC varied between 1.73–2.0 and 1.52–2.66 µmole TE/mg DW in white and brown genotypes, respectively, with no difference between the genotypes (Table 1).

**Figure 3:**
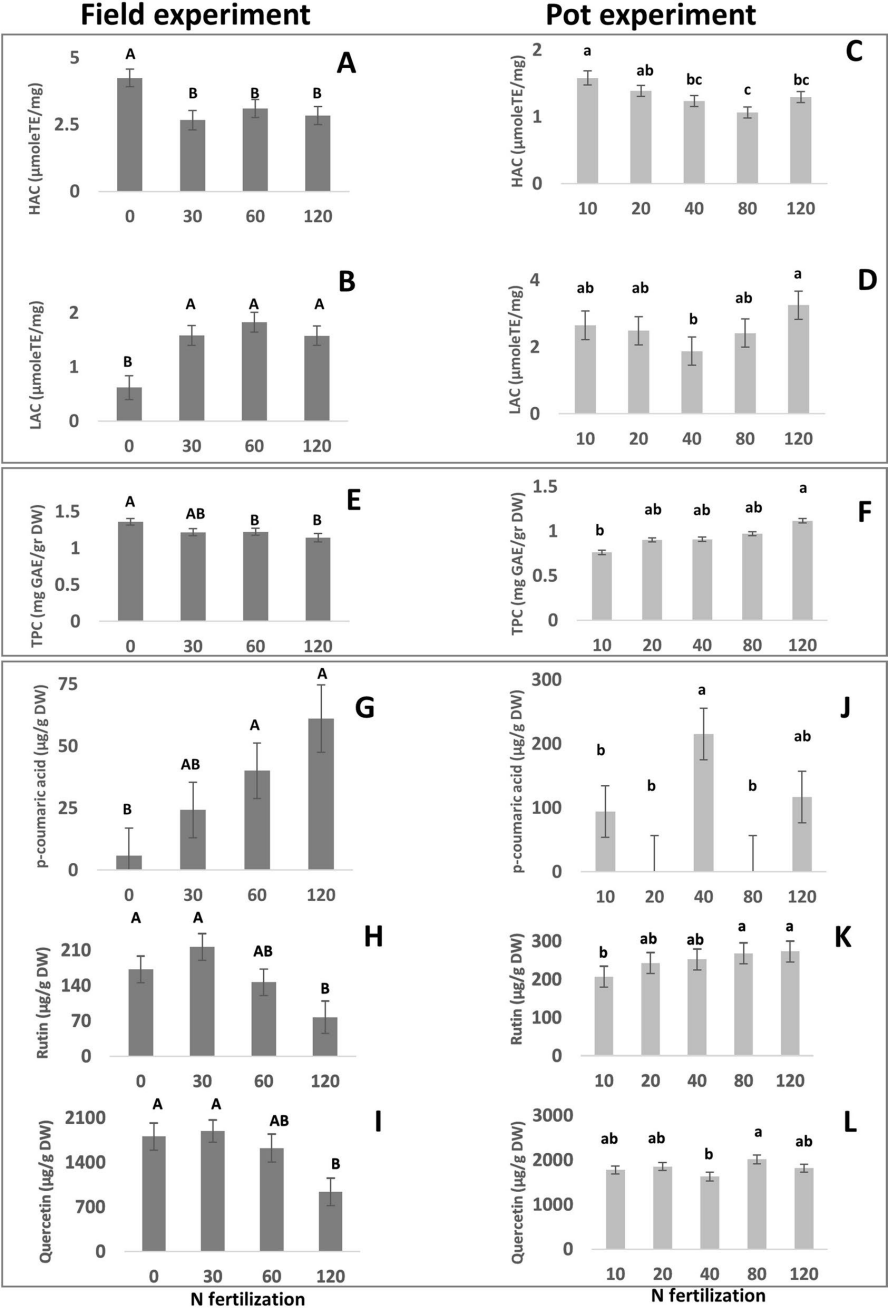
Effect of nitrogen (N) treatments on various nutritional quality traits of tef grains, in field (dark bars **A**,**B**,**E**,**G**–**I**) and pot (light bars; **C**,**D**,**F**,**J**–**L**) experiments. All figure results are presented as mean ± SE.

In the field experiment, hydrophilic antioxidative capacity (HAC) significantly decreased with increasing N fertilization (4.24 µmole TE/mg DW in 0 ppm to 2.64, 3.1 and 2.83 µmole TE/mg DW in 30, 60 and 120 ppm, respectively; Fig. 3A), while lipophilic antioxidative capacity (LAC) significantly increased from 0.62 µmole TE/mg DW to 1.58, 1.58 and 1.8 µmole TE/mg DW in 30, 60 and 120 ppm N (Fig. 3B).

In the pot experiment no effect on hydrophilic or lipophilic antioxidative capacity was observed (Fig. 3C,D, respectively).

### Free, bound and total phenolic content

Total phenolic content (TPC), as well as free and bound phenolics content of tef grains have been published previously^43^. In the current study TPC ranged between 0.89–1.2 and 1.04–1.27 mg GAE/gr DW in grain of the white and brown genotypes, respectively (Table 1), with brown genotype showing higher content than the white genotype in both experiments (Table 1). Free phenolic content in this experiment was 2.02 to 2.24 and 2.02–2.14 mg RE/g DW in white and brown genotypes, respectively, higher than the content of bound phenolics, which was 0.25–0.73 and 0.37–0.93 mg RE/g DW in the white and brown genotypes, respectively, and did not differ between genotypes (in both field and pot experiments).

TPC decreased with increasing N in the field experiment, from 1.36 mg GAE/gr DW to 1.2 and 1.14 mg GAE/gr DW in 30 and 120 ppm, respectively (Fig. 3E). Free and bound phenolic content was unaffected by N (Table 2).

In the pots, TPC increased with increasing N (0.76 mg GAE/gr DW at 10 ppm vs 1.16 mg GAE/gr DW in 120 ppm; Fig. 3F), while free and bound phenolics levels were not affected by N (Table 3).

### Phenolic profile

Phenolic profile of tef comprises mainly of phenolic acids, in addition to flavonoids^43^. In our samples we were able to identify six phenolic compounds, including three phenolic acids: genistic, p-coumaric and ferulic, two flavonoids: rutin and quercetin, and phenolic aldehyde, vanillin. The most abundant phenolic compounds in our tef samples were the flavonoids quercetin (1,403.7 to 1,607.8 and 1,429.5 to 1,486.2 µg/g DW in the white and brown genotypes, respectively), and rutin, (146.3–262.9 and 125.6 to 189.1 µg/g DW). These were followed by the phenolic acids genistic (19.5 to 88.6 and 19.7 to 126.1 µg/g DW), p-coumaric (46.0 to 165.4 and 3.92 to 19.5 µg/g DW) and ferullic (19.3 to 79.6 and 75.9 to 126.1 µg/g DW), and vanillin (16.5 to 37.9 and 157.6 to 174.7 µg/g DW) (Table 1). The white genotype had higher levels of p-coumaric in both experiments, and higher level of rutin in the pot experiment, while the brown genotype had higher levels of vanillin and ferulic acid in both experiments.

In the field experiment, a significant increase was observed in the contents of p-coumaric acid with increasing N levels, from 5.83 µg/g DW in 0 ppm to 40.0 and 61.0 µg/g DW in 60 and 120 ppm (Fig. 3G), with a decrease in the contents of both rutin (172.0 and 216.3 µg/g DW in 0 and 30 ppm N to 77.0 µg/g DW in 120 ppm; Fig. 3H) and quercetin (from 1803.9, 1886.8 and 1621 µg/g DW in 0,30 and 60 ppm, respectively, to 934.2 µg/g DW in 120 ppm; Fig. 3I).

In the pots, p-coumaric acid levels were only very slightly affected by N fertilization, with 40 ppm treatment (215 µg/g DW) higher than 10, 20, 80 and 120 ppm (94.3, 0, 0 and 116.8 µg/g DW, respectively; Fig. 3J). An increase in rutin was observed with increasing N levels, from 206.5 µg/g DW in 0 ppm to 272.7 µg/g DW in 12 ppm (Fig. 3K), with only a slight impact on quercetin (higher level of 2014.9 µg/g DW in 80 ppm treatment compared to 1629 µg/g DW in 40 ppm; Fig. 3L).

## Discussion

N effect on crop performances and specifically quality parameters, has been described^40,41^, often for common commodities, e.g. potato, tomato, apple etc. At the same time, the results of the current work will be mainly compared to those reported on cereal, as these are more relevant to tef. Although millets and sorghum would make the best comparison, most of the available data refers to wheat grains, with some data on rice. Additionally, in this work tef grains from two growing platforms were tested-field plots and pots in greenhouse. It is generally accepted that both field and greenhouse results are specific to given environment and genetic background. However while the pot results reflect the crop potential, as the growing conditions are well controlled, the field results are more relevant to practical conditions. Furthermore, the field and pot experiments took place in different seasonal conditions, since seeds were sawn in different dates (see “Materials and methods” section). Therefore, their growth season was different, which can explain some of the variability in the response to N fertilization. Additionally, different response to N fertilization in perlite (pots) in comparison to soil originates in other factors, e.g. differences in ion exchange, water availability. Although in many cases the results were similar for both platforms, in some other they differed in either values or effect trends. However, it is important to mention that no contradiction was found within the two dataset. In addition, due to the different nature of these platforms, no attempt was done to statistically compare them, as such comparison will not be informative due to the wide array of possible variability sources.

The mineral content of tef grain is higher than that of most staple cereals used in western nutrition, with high levels of Ca, Fe, Cu, Mg and Zn^9^. Although mineral composition of tef grains has been published, only a few reports show detailed data regarding both white and brown tef genotypes, with some only specifying a general range of values. Our results were generally in agreement with previously published data, with some differences between genotypes. For Ca, levels of 124 and 155^6^ or 17–124 and 18–178 mg/100 gr^25^, were reported for white and brown tef grains, respectively. Our results were in this range in the pot experiment, and slightly higher in the field experiment, with no apparent difference between genotypes. For Mg, this work is the first to present contents of both white and brown tef, which is in agreement with available data of 184–200 mg/100 gr^13^, with white genotype showing higher levels than brown in the field. Fe levels were reported as 37.7^6^, 31.6^44^ or 15.9 mg/100 gr^45^ and 24.6 mg/100 gr^45^ for white and brown grains, respectively. Previously reported high levels of above 150 mg/100 gr can probably be attributed to soil contamination, as suggested^6^. Our results are slightly below this range, with no difference between genotypes. Zn content was reported as 2.86–4.02^6^ and 2.4–6.8 and 2.3–6.7^25^ mg/100 gr in white and brown tef, respectively, and our results are well within this range for both genotypes in the field, and higher in white genotype compared to brown, and slightly higher in the pots. For Mn content, this is the first report on different genotypes, and in the field results both genotypes were in the range of values published for tef of 56.5 ppm^11^ and 3.8 mg/100 gr^9^, in the pot experiment both genotypes showed higher levels, with brown genotype higher than white. As for Cu, white and brown genotype content was reported as 2.5–5.3 and 1.1–3.6 mg/100 gr, respectively^25^. However, we found much lower levels, with white genotype containing higher levels than brown in the field.

Grain Zn levels in wheat were reported to positively respond to N supply when Zn levels in soil and tissues are sufficient, although results in rice showed that this effect depends on initial seed Zn levels and yield capacity^46^. This might explain the observed mixed trend of a decrease in the field plants, alongside the increase in the pot plants. For Fe, N fertilization was reported to positively affect both acquisition and grain allocation in wheat^47^, showing the same trend as in our data. Likewise our results, Mn was reported to remain unchanged in wheat grains in response to increasing N. Cu was reported to increase in wheat grains^48^ but decrease in rice^49^. Likewise our data, Mg levels decreased and Ca increased^49^. There seems to be an agreement across works in various cereal regarding the increase in Zn and Fe with increasing N, however in regard to other minerals published data is generally inconsistent, thus implying that other genetic and environmental factors are involved in plant mineral content responses to N fertilization.

In this work, we report for the first time a detailed fatty acid composition of tef grains, for two genotypes. Lipids in tef are nutritionally important since they play a role in baking-related qualities, binding to gluten/non gluten proteins and affecting the bioaccessibility of polyphenols in bread^50^. Fatty acids are also important for sensory properties of the final baked products, contributing to texture and taste. In addition, as tef is fermented to make injera, initial fatty acid composition of the fermented substance may affect its fermentation properties, as was reported for other fermented foods, e.g. beer^51^ and olives^52^.

Tef fatty acid profile generally resembles that of other cereal, with the PUFA C18:2 as the main fatty acid, followed by C18:1 and C16:0^53^, and trace amounts of longer C20:0 and C22:0 fatty acid like other millets^54^ and quinoa^55^. Two available reports only generally described fatty acid composition of white tef grains, showing slightly different profiles: an older work^14^ showed that oleic acid content (32%) was higher than that of linoleic acid (24%), while our results are in agreement with those of Hager et al.^42^, where linoleic acid content (50%) is higher than oleic acid (29.5%)^42^. High levels of unsaturated fatty acids are nutritionally desirable, due to their positive health effects^42^. Specifically, the presence of linoleic (C18:2) and α-linolenic (C18:3) acids is valuable, being essential fatty acids not synthesized by the human body. Tef profile is unique in containing higher levels of C18:3, in addition to odd-chain fatty acids, C15:0, C17:0 and C21:0, not commonly found in nature. Consumption of odd fatty acids was correlated with unfavorable health effects^56^. Nevertheless, these fatty acids can be utilized by bacteria through the a-oxidation pathway^56^, and thus fatty acid composition may be important for the fermentation process. Since non-digestible components of the cereal matrix may also serve as prebiotics^57^, these might also contribute to tef gut-microbiota benefits, working either as a pre or probiotic substance.

Although fatty acid amount might seem small and non-significant, cereals actually make a significant contribution to essential fatty acid consumption, being consumed in large amounts^58^. Throughout our data, the white genotype was consistently higher in C18:0 and C20:0, while the brown genotype had higher levels of C18:3 and PUFA (Fig. 1). Nutritionally, the consumption of saturated fatty acids such as C18:0 and C20:0 is undesired, correlating with adverse effects such as heart disease and metabolic syndrome, while consumption of PUFA, and mainly C18:3, is recommended to maintain good diet and health. Thus, although from a cultural point of view white tef is preferred over brown tef, from a nutritional point of view brown tef seems to be superior in regard to fatty acid composition, presenting a higher contents of essential and health-beneficial acids. Additionally, white tef may be preferred due to the presence of higher levels of more palatable saturated fatty acids.

Antioxidative capacity (AOC) is an important health-related trait of foods, reflecting not only the chemical and phytochemical composition, but also the biological activity. Much data is available regarding antioxidative capacity of cereals in general and of tef, however not much of it address the differences between white and brown tef genotypes. In addition, this work shows for the first time the hydrophilic and hydrophobic AOC of tef. Antioxidative capacity of cereals and millets was reported, showing 0.5–0.9 µM TE/g for rice and amaranth, 1.4 for quinoa and 2.4 µM TE/g for buckwheat^59^, 8.5 µmol TE/g for wheat, 15 for barley, 13 for rye, 21.4 for pearl millet and 52 µmol TE/g for sorghum^60^. In different reports, white and brown tef total AOC was found to be 2.9–3 and 4.6–6 µM TE/g^43^, 40 and 50 µM TE/g^22^ and 9.3 and 10.3 mmol TE/kg^61^, respectively, and 35 µmol TE/gr for brown tef^62^. Clearly, not all sources are comparable, however when summing up our HAC and LAC for calculation of total AOC, values are in accordance with other works, which show that when compared to other staple western cereals and gluten free cereals and millets, tef has a higher AOC than most^62,63^.

High levels of N fertilization was reported to decrease AOC in wheat^64^, similarly to our HAC field results. HAC is the main antioxidative capacity (AC) in tef, being higher than LAC, implying that most of the AC in the grain originate in hydrophilic compounds, e.g. polyphenols, rather than lipophilic antioxidants, e.g. tocopherols, as was demonstrated for tef^43^. In addition, in the field experiment N fertilization response trends were similar for HAC and TPC, and correlated well (R^2^ = 0.40, *p* < 0.012), which may imply that polyphenolic compounds are at least partially attributed to the antioxidative activity in tef.

In comparison to other cereal, tef is relatively rich in phenolic compounds^22,62^. TPC values of tef grains are presented in several works: 1.4 to 1.6 mg GAE/g for white and 1.9–2.2 mg GAE/g for brown tef^43,61^, 263–500 mg catechin equivalent (CE)/100 gr in white and 409–700 mg CE/100 gr in brown^22,65^. Our results are in agreement with previous data, i.e. within the same range and follow the clear trend of higher TPC values for brown vs white tef genotypes as shown in other works, and in general for pigmented cereal^43^. As this is the first report presenting the phytochemical and nutritional composition of tef grains grown in Israel, it was of interest to compare current results to existing information regarding teff grain health-related composition.

Free and bound phenolics in white tef range 0.9–1.2 and 0.4–0.5, respectively, and 1.4 and 0.5–0.8, respectively, in brown genotypes^43^. Our field and pot results for both genotypes are within this range. It is important to mention that free and bound phenolics do not sum up to total phenolics measurement due to different extraction methods.

TPC, as well as the content of specific polyphenols, was reported to decrease in wheat in response to high N fertilization^64,66^, which was hypothesized to result from stress condition imparted by high N levels, thus consuming polyphenols to scavenge the resulting reactive oxygen species (ROS)^64^. Nevertheless, some other works reported an increase in wheat TPC in response to N availability^67^. Interestingly, It was postulated that while free soluble phenolics increase with increasing N supply, conjugated soluble compounds decrease, and bound forms are not affected^66^. The invers trends observed in the field vs. pot experiments may reflect a different TPC composition of the grains, i.e. higher levels of conjugated phenolics in the field and higher levels of free phenolics in the pots. In agreement with mentioned report, bound phenolics were not affected in both experiments.

A detailed phenolic profile of tef have been published for both grain colors, and includes flavonoids, stilbenes and phenolic acids^22,43,61,65,68,69^. In this work, we focused on the main free polyphenolic compounds, and our results are concomitant with those of Kotaskova and co-workers^43^, showing that the white genotype was higher in rutin, while the brown genotype higher in ferulic acid (Table 1). Among the compounds we detected, quercetin and rutin were the major phenolic compound, with much higher levels compared to other reports. However, since genotype and environmental conditions greatly affect polyphenolic profile^43^, these may be among the major reasons for the observed difference between our results ad previous reports. In the same manner, we were also able to identify vanillin in our samples, not previously reported in tef. These phenolic compounds are highly abundant in nature, and many cereals present a similar phenolic profile^70^, with ferulic acid as a major phenolic acid^71^. Furthermore, the trends for rutin and quercetin response to N fertilization was similar to that of TPC in both filed and pot experiments, which might support their presence as major phenolic compounds in our samples (Fig. 3).

Numerous reasons may account for the observed variety effects, including inherent genetic variation in N-use efficiency as was previously reported for tef^33,36^, possibly due to a rhizobacteria effects, as was indicated for wheat^72^ and tef^73^. Moreover, tef varieties may differ in N-utilization through biosynthetic pathways, e.g. grain production, as implied by differences in their yield^74^. As for possible reasons for observed N effects, N fertilization has been reported to affect mineral accumulation in grain through remobilization of micronutrients within plants , influencing the translocation of metals like Fe and Zn^48^. N availability was also reported to affect fatty acid synthesis, although no mechanism has been suggested^75^. Polyphenolic compound biosynthesis is also affected by N, through increasing amino acid content, including those which are precursors of phenolic acids^41,76^.

As for the possible dilution effect of high yield on reducing seed quality, in our experiments trends of quality parameter levels were not concomitant with those of yield^74^. We thus assume that the possible negative effect of high yield on quality was non-significant under these conditions.

In conclusion, interest in tef is increasing worldwide thanks to its beneficial health effects and gluten-free properties. In this work, we show for the first time the effect of N availability on brown and white tef grain, in addition to a detailed phytochemical profile of both genotypes. The results presented here can be implied as part of a biofortification tool for functional food, aiming at producing healthier and more nutritional food by means of agrotechnology rather than by addition of artificial additives.

While fertilization is crucial for crop managements and high yield, it also affects nutritional value of the food. N fertilization affects tef health and nutritional value, including mineral content, fatty acid profile, anti-oxidative capacity and polyphenol levels and composition. These effects should be considered when deciding on fertilization regime, to optimize both plant physiology, productivity and food-related effects. Of specific consideration is Fe and Zn content, since many health-aware consumers who consume tef and are vegetarian or vegan, low in these nutrients. In addition, being gluten-free, tef in consumed in large amounts by celiac patients, who already have a problematic mineral absorption due to colon inflammation, and if N fertilization management lowers the mineral content this should be noted and acknowledged. At the same time, it should be mentioned that conclusions from the current study are naturally limited, the results being based on only a 1-year field trial and one greenhouse experiment, and are not always consistent for some of the nutritional parameters (Tables 2, 3). Hence, more research is required in order to elucidate the effects of crop cultivar and management on tef grown under irrigation in a dry region like Israel.

It is also important to note that that in addition to the nutritional quality traits we chose to evaluate in this work, there are also some very important organoleptic quality characteristics, including taste, aroma and texture of the final fermented product (e.g. injera bread). In addition, other nutritional aspects may also play a role in quality, i.e. protein and fiber content, as well as the presence of anti-nutrients previously reported in tef, e.g. phytate.

## Materials and methods

### Chemicals

All chemicals and commercial standards were purchased from Sigma (Sigma, St. Louis, MO, USA). Acetone, methanol, hexane, acetic and hydrochloric acid were from BioLab (BioLab, Jerusalem, Israel). Ethanol was from Gadot (Gadot, Netanya, Israel). Rutin and trolox were purchased from Acros Organics (Acros Organics, New Jersey, USA). Lanthanum chloride was from EMD (Millipore Darmstadt, Germany).

### Plant material

Brown (405B) and white (406 W) teff seeds were obtained from The Israeli Gene Bank and grown in Gilat research Center in the northwestern Negev, Israel (31° 20′ N, 34° 41′ E). Plants were grown to full maturity and harvested manually.

### Pot experiment

Pot experiment was conducted in a walk-in plastic-covered tunnel (6 m wide, 2.4 m tall and 30 m long), during winter 2015–2016. The experiment comprised of five treatments of N concentrations in the irrigation solution: (10, 20, 40, 80 and 120 ppm) and two teff genotypes. A factorial (N treatments × genotypes) completely randomized block design was employed with five replicates. In all treatments, N was provided as 90% NO_3_^−^ and 10% NH_4_^+^. The teff plants were grown in 3L pots containing perlite as a growth medium. Seeds of the two teff genotypes were sown on 15 December 2015, minimum temperatures during the experiment ranged between 0 and 16 °C and maximum temperatures were 16–35 °C. Initially, the all pots were drip irrigated with a solution containing 40 ppm N, 6 ppm P, 40 ppm K, 20 ppm Ca, 20 ppm Mg, 28 ppm S, 0.3 ppm B, 0.6 ppm Fe, 0.3 ppm manganese (Mn), 0.15 ppm Zn, 0.02 ppm Cu, and 0.02 ppm Mo. Thinning was carried out 3 weeks after emergence and 15 plants per pot remained for the entire experiment. Twenty five days after sowing, differential N treatments were initiated, with all other nutrients kept throughout the entire experiment at their initial concentrations. Pots were irrigated with final solutions, according to treatments, via a drip system. In the pot experiment, the yield recorded for the white variety was 10–25 gr/pot, while the brown variety yielded 8–16 gr/pot. Detailed experimental information for both field and pot is described by Gashu et al.^74^.

### Field experiment

Field experiment was conducted during summer 2016. The soil type was a typic Haploxeralf, sandy loam loess, containing 55% sand, 30% silt and 15% clay, and initial soil N availability was 0.18, 0.34 and 0.25 mg/L NO_3_ at 0–30, 30–60 and 60–90 cm respectively, and 3.03, 0.9 and 0.72 mg/kg soil of NH_4_ at 0–30, 30–60 and 60–90 cm, respectively. Minimum temperature during the experiment ranged between 12 and 22 and maximum temperatures were 29–39 °C (min/max), with no rain events recorded. Experiment included four N levels: (0, 30, 60 and 120 ppm in the irrigation water). N was provided as 70% NO_3_^−^ and 30% NH_4_ in all treatments_._ Concentrations of all other minerals were identical to those used in the pot experiment. A factorial (N treatments × genotypes) split plot block design was used. Each plot (5 m × 2.1 m) consisted of 28 rows (14 rows per genotype). Each main plot was irrigated by 14 drip lines (one line between each pair of rows). Seeds were directly sown on 13 July 2016 into well-prepared dry soil at a depth of ~ 1 cm. Two weeks after sowing, fertigation treatments were started by injecting 1L of custom-made fertilizer solutions to 100 L of water. Fertigation was applied daily via a drip system. In the field experiment, the yield recorded for the white variety was 26–61 gr/m^2^, while the brown variety yielded 79–121 gr/m^2^^74^.

### Seed sample preparation

For sampling, three replicates of 5 gr. seeds each from each treatment were freeze-dried in a lyophilizer (Martin Christ, Germany). The dry seeds were powdered by a bead-beater (Zeleniki, Slovenia) at 30 Hz for 1 min with two 1 mm stainless steel beads, and the powder was kept at −20 °C.

### Mineral content

For mineral content determination, 1 gr of lyophilized grain powder was baked at 350 °C for 60 min and then at 550 °C for another 5 h. After cooling, 5 ml of HCl was added, and after 60 min the samples were filtered through 42 Whatman filter paper, and 20 ml DD water was added, to a total volume of 25 ml. One ml of lanthanum chloride 98% solution was added to 0.1 ml of the solution, and brought to a final volume of 10 ml with DD water. Minerals were quantified by atomic absorption spectrometry (Perkin Elmer Precisely Analyst 200).

### Fatty acid composition

Fatty acid profiling by gas chromatography was performed as we described earlier^77^. The relative composition of fatty acids was determined as percentage of total fatty acid content.

### Antioxidative capacity

For evaluating hydrophilic and lipophilic AO capacity of tef grains, 2,2′‐azino‐bis(3‐ethylbenzothiazoline‐6‐sulfonic acid) (ABTS)- Trolox equivalent antioxidant method was employed, as described earlier^78,79^.

### Free and bound phenolic content

Free and bound phenolic content was measured according to the method described by Kotaskova et al.^43^. For free phenolics, 0.1 gr lyophilized tef seed powder was weighed in an Eppendorf tube, and 500 µl of 75% (v/v) acetone in water solution were added, and centrifuged for 5 min at 17 KG at room temperature, three times. The supernatant was pooled and brought up to a final volume of 1.5 ml. For bound phenolic content, the residues after the free phenolic extraction were dried using a speed-vac (Gemini BV, Netherlands) for 15 min at 15 mbar. 1.5 ml of 2 M NaOH were added the dried residue and vortexed for 30 s, and then mixed at a thermoshaker (AccuTherm, NJ, USA) at 70 °C, 500 rpm for 70 min. 200 µl of the extract were read in a 96-well plate on 360 nm (wavelength optimized to solvent using rutin).

### Total phenolic content

Total phenolic content was measured as described earlier^78^, modified to 96 well plates. Briefly, 0.1 gr of tef seed powder was weighed and 500 µl of 80% ethanol solution (v/v) were added, mixture vortexed and centrifuged for 5 min at 17 KG at room temperature, three times. Supernatant was pooled and brought up to 1,500 µl. 13 µl of the extract were then added to 750 µl double distilled H_2_O (DDW) and 63 µl Folin-Ciocalteu reagent and vortexed. 188 µl of 20% Na_2_CO_3_ (v/v) and 238 µl of DDW were added and the mixture was let stand for 75 min, after which 200 µl were read in a 96 well plate at 765 nm in a spectrophotometer. Gallic acid (0–600 mg/l) was used as a standard and the results were expressed as mg of gallic acid equivalent (GAE)/g DW of the sample.

### Basic polyphenol profile

Polyphenols were extracted from 0.3 gr of tef powder by adding 1 ml of 80% ethanol solution (v/v), mixing, centrifuging (17 KG for 5 min). The extract was filtered through 0.45 µm polytetrafluoroethylene (PTFE) filter and 1.5 ml were aliquoted in a vial. Basic polyphenolic profiling of tef seeds was done using an Ultra Performance Liquid Chromatography (UPLC) system (ACQUITY UPLC H class, Waters, Millford, MA, USA), consisting of a photo diode array (PDA) detector, vacuum degasser, an auto sampler, a binary pump and a reversed phase Benzene hexachloride (BHC) C18 analytical column (2.1 mm × 100 mm, 1.7 µm, Waters). The mobile phases consisted of Milli Q water (0.1% formic acid, v/v) (A) and 100% methanol (UPLC grade) (B). Flow rate was 0.3 ml/min and column temperature maintained at 35 °C. The program was as follows: 1 min at 98% A, decreasing to 95% A over 1 min, decreasing to 30% over another 5 min, further decreasing to 5% A over the next 3 min and final decrease to 0% A over the next 2 min, followed by an increase to 95% A over another 2 min and re-equilibrate to 98% A over the last minute. Identification and quantification of quinic, gallic, protocathchic, caffeic, vanillic syringic, trans-cinnamic, genistic, ferulic, and p-coumaric acids, vanillin, rutin, catechin and quercetin was done with commercial standards. For identification, multiple reaction monitoring (MRM) was used in positive and negative mode, in 5–1,200 mass range, and the MS data was processed by Masslynx software (Waters, Millford, MA, USA). Quantification was performed using calibration curves based on PDA. Wavelength and MRM information for each standard are presented in Table S1.

### Statistical analysis

Statistical analysis was performed by JMP 13. For Tables 1, 2 and 3 a multifactorial model using analysis of variance (ANOVA) was used, with N fertilization level (continuous) and genotype (character) as predictor variables. For Figs. 1, 2 and 3 a second analysis was performed, using a different model, defining N as ordinal and as a single predictor variable, followed by Tukey pairwise comparisons. The resulting treatment level means are presented as bar graphs, in order to show specific levels of quality parameters in the grains as affected by fertilization. Only statistically significant results are presented and discussed, thus all the results mentioned are statistically significant. Where N × genotype interaction was not significant, the values are presented for genotype averages (Table 1).

## Supplementary information

Supplementary Table S1.
