# Supplementary material for: Nitrogen availability and genotype affect major nutritional quality parameters of tef grain grown under irrigation
Source: Sci Rep. 2020 Aug 31;10:14339. doi: 10.1038/s41598-020-71299-x (PMC7459322; doi:10.1038/s41598-020-71299-x)
Supplement: Supplementary file 1 — Supplementary Table S1. [file 41598_2020_71299_MOESM1_ESM.docx]

**Nitrogen availability and genotype affect major nutritional quality parameters of tef grain grown under irrigation**

Zipora Tietel^a*^ Ella Simhon^a,b^, Kelem Gashu^c,d^, Arul Annanth^a^, Betty Schwarz^b^, Yehoshua Saranga^c^ Uri Yermiyahu^d^

| **Sl. No** | **Analyte** | **tR (min.)** | **MW (g/mol)** | **λ max (nm)** | **MRM transition** | | **CE** | **CV** |
| --- | --- | --- | --- | --- | --- | --- | --- | --- |
|  |  |  |  |  | **Parent ion** | **Product ion** |  |  |
| 1 | Quinic acid | 0.87 | 192.17 | 230 | 191.00 | 84.60 | 24 | 44 |
| 2 | Gallic acid | 1.80 | 170.12 | 272 | 168.10 | 96.90 | 22 | 40 |
| 3 | Protocatechuic acid | 3.40 | 154.12 | 260 | 152.90 | 108.90 | 14 | 32 |
| 4 | Gentisic acid | 3.71 | 154.12 | 262 | 153.00 | 107.90 | 20 | 8 |
| 5 | Catechin | 3.80 | 290.26 | 279 | 291.17 | 77.00 | 12 | 16 |
| 6 | Caffeic acid | 4.04 | 180.16 | 240 | 179.07 | 106.90 | 22 | 40 |
| 7 | Vanillic acid | 4.12 | 168.14 | 260 | 167.00 | 107.90 | 14 | 36 |
| 8 | Syringic acid | 4.13 | 198.17 | 276 | 197.08 | 94.90 | 22 | 38 |
| 9 | Vanillin | 4.33 | 152.12 | 280 | 151.07 | 135.00 | 10 | 26 |
| 10 | *p-*Coumaric acid | 4.54 | 164.04 | 228 | 163.93 | 93.54 | 28 | 24 |
| 11 | Ferulic acid | 4.63 | 194.18 | 324 | 193.09 | 133.90 | 14 | 42 |
| 12 | Rutin | 4.90 | 610.52 | 258 | 609.24 | 301.20 | 38 | 46 |
| 13 | Quercetin | 6.24 | 302.24 | 260 | 301.07 | 151.00 | 20 | 10 |
| 14 | *trans-* Cinnamic acid | 6.49 | 148.16 | 274 | 146.95 | 102.90 | 10 | 44 |

**Table S1**. Polyphenol mass spectrometry (MS) analysis parameters. tR- time of retention; MW- molecular weight; λ- wavelength; MRM- Multiple reaction monitoring; CE- collision energy; CV- collision voltage.
